# Natural Products in Preventing Tumor Drug Resistance and Related Signaling Pathways

**DOI:** 10.3390/molecules27113513

**Published:** 2022-05-30

**Authors:** Chuansheng Yang, Zhikai Mai, Can Liu, Shuanghong Yin, Yantao Cai, Chenglai Xia

**Affiliations:** 1 Department of Head-Neck and Breast Surgery, Yuebei People’s Hospital of Shantou University, Shaoguan 512027, China; ychssmile@163.com; 2 Affiliated Foshan Maternity and Chlid Healthcare Hospital, Southern Medical University, Foshan 528000, China; mzk0921@163.com (Z.M.); liucan178@163.com (C.L.); 15307334102@163.com (S.Y.); 3 School of Pharmaceutical Sciences, Southern Medical University, Guangzhou 510515, China

**Keywords:** tumor, drug resistance, signaling pathway, mechanism

## Abstract

Drug resistance is still an obstacle in cancer therapy, leading to the failure of tumor treatment. The emergence of tumor drug resistance has always been a main concern of oncologists. Therefore, overcoming tumor drug resistance and looking for new strategies for tumor treatment is a major focus in the field of tumor research. Natural products serve as effective substances against drug resistance because of their diverse chemical structures and pharmacological effects. We reviewed the signaling pathways involved in the development of tumor drug resistance, including Epidermal growth factor receptor (EGFR), Renin-angiotensin system (Ras), Phosphatidylinositol-3-kinase/protein kinase B (PI3K/Akt), Wnt, Notch, Transforming growth factor-beta (TGF-β), and their specific signaling pathway inhibitors derived from natural products. This can provide new ideas for the prevention of drug resistance in cancer therapy.

## 1. Introduction

Tumors continue to be a threat to human health, despite the many therapies available to treat malignant tumors. At present, the treatment of tumors includes chemotherapy, radiotherapy, surgical treatment, and immunotherapy [1]. Although tumor treatments are constantly improved, chemotherapy remains the most common method of tumor treatment [2]. However, in the process of using combination or single targeted chemotherapies, there are many drug-related side effects [3]. As treatment continues, tumors often become resistant to chemotherapeutic compounds. Currently, tumor-targeted inhibitors and cytotoxic drugs use one or several specific biomolecules to activate corresponding signaling pathways and exert antitumor effects. Thus, tumor resistance generation is associated with the reactivation or inhibition of these signaling pathways. The mechanisms of tumor resistance are complex [4]. Resistance is primarily manifested in the following ways [5]. (1) Tumor cells can modify tumor suppressor targets or their downstream signaling pathway proteins through the endocrine pathway to generate tumor drug resistance [6]. (2) Tumor cells may have enhanced DNA repair capacity, conferring resistance to platinum compounds or poly (ADP-ribose) polymerase (PARP) inhibitors [7]. (3) Loss of tumor-specific antigens can lead to resistance to immunotherapy [8]. (4) ATP-binding box transporter family members, ATP-binding cassette superfamily B member 1 (ABCB1), ATP-binding cassette subfamily C member 1 (ABCC1), and ATP-binding cassette transporter of subfamily G members (ABCG2) promote drug efflux, leading to tumor drug resistance [9]. (5) Cancer stem cells (CSCs) have a low division rate, which can upregulate the expression of ATP binding box transporter family members, improve DNA repairability, and induce tumor drug resistance [10]. (6) When cell polarity and cell contact are lost in epithelial cells, specific epithelial markers (such as E-cadherin and cytokeratin) are reduced, and mesenchymal characteristics (such as increased cell motility, vimentin, N-cadherin, fibronectin, and matrix metalloproteinase) increase, activating ABC transporters and regulating anti-apoptotic pathways, thus participating in tumor resistance to cytotoxic drugs, tyrosine kinase inhibitors, and endocrine therapy [11]. 

Certain signaling pathways play important roles in the development of drug resistance. For example, the epidermal growth factor receptor (EGFR) signaling pathway can activate the Ras/Raf/mitogen-activated protein kinase (MEK)/extracellular signal-regulated kinase (ERK) or phosphatidylinositol 3-kinase (PI3K)/Akt/mammalian target of rapamycin (mTOR) cascades to regulate cell growth, differentiation, adhesion, and migration [11,12]. Abnormal regulation of EGFR signaling is closely related to drug resistance in cancer [13]. Studies have reported that mutant EGFR can activate the mitogen-activated protein kinase (MAPK) signaling pathway and intersect with the Akt pathway to induce non-small cell lung cancer (NSCLC) resistance to AZD929. When MAPK signaling is activated, lung cancer patients with fibroblast growth factor receptor (FGFR)1 mutations can become resistant to FGFR inhibitors [14]. MEK is a component of the Ras signaling pathway, and when MEK is activated, the EGFR-Ras-Raf signaling pathway is activated, which can eventually lead to tumor resistance to MEK inhibitors [15]. MEK activates the ErbB3 signaling pathway, leading to gefitinib resistance in lung cancer [16]. In addition to EGFR activation of Ras signaling, EGFR activation of PI3K signaling also plays an important role in inducing drug resistance in tumors [17]. PI3K signaling was found to be activated in acute leukemia cells resistant to chemotherapeutic drugs. When PI3K and MAPK pathways are jointly activated, melanoma can induce the resistance of Raf mutations to MAPK inhibitors [18]. Other signaling pathways, such as the Wnt signaling pathway, can also induce the generation of tumor drug resistance [19]. It was found that abnormal Wnt signaling can upregulate ABCB1 and ABCG2, inducing NSCLC resistance to cisplatin [20]. Furthermore, notch signaling, by upregulating ABCB1, can induce doxorubicin resistance in prostate cancer cells [21]. The inhibition of Notch-1 signaling restores the sensitivity of breast cancer cells and prostate cancer cells to doxorubicin and docetaxel [22].

Chemotherapy is a conventional treatment for cancer. However, cancer cells are resistant to almost all types of chemotherapeutic and targeted drugs, and approximately 80–90% of deaths in cancer patients are directly or indirectly attributed to resistance, making it a considerable challenge in cancer treatment [23]. Fortunately, natural products with different chemical structures and pharmacological effects are effective substances against drug resistance [24]. Natural products reverse drug resistance by regulating proteins involved in resistance, targeting non-apoptotic cell death, and inducing other types of non-apoptotic cell death [25]. Many natural products have very strong drug resistance reversal properties, among which are alkaloids, flavonoids, phenylpropanoids, and terpenoids (Table 1). For example, matrine can improve the sensitivity of resistant cancer cells to chemotherapeutic drugs by reactivating apoptosis and inhibiting drug efflux [26]; Tetrandrine can fight the multiple-drug resistance (MDR) of cancer cells by downregulating the expression of ABCB1 transporters [27]; quercetin can reverse the resistance of paclitaxel-resistant prostate cancer cells in vitro by reversing activation of the androgen receptor and PI3K/Akt signaling [28]. Osthole reverses the chemotherapeutic resistance of cisplatin-resistant cervical cancer cells by inactivating the PI3K/AKT pathway [29]; and ginsenoside Rg3 can inhibit the mTOR signaling axis of SOX2, PI3K/Akt, and miR-429 to reduce the resistance to cisplatin [30]. 

Here, we reviewed the molecular mechanisms of tumor drug resistance induced by EGFR, PI3K/Akt, Ras/MAPK, Wnt/β-catenin, Notch, Transforming growth factor-beta (TGF-β), and combinations of these signaling pathway inhibitors derivatives from natural products to overcome tumor resistance, provide new ideas and strategies for the rational clinical selection of antitumor drugs, and therefore improve patient quality of life and survival.

## 2. The Signaling Pathways in Tumor Drug Resistance

It has been well established that signaling pathways interact to form complex regulatory networks. Signaling pathways are a key factor in maintaining the homeostasis of the intracellular environment [31]. Many signaling pathways play important roles in tumor transformation, metastasis, inhibition of apoptosis, and tumor stem cells [32,33]. Abnormal activation or inhibition of one or more signaling pathways can lead to tumorigenesis or induce drug resistance [34]. At present, chemotherapy remains the main treatment for tumors [35]. Mutations of the targets of chemotherapeutic drugs, or modifications of the signaling pathway components involved, can reduce drug efficacy so that the tumor develops resistance to the chemotherapeutic drugs [36]. The mechanisms of tumor resistance to chemotherapeutic drugs are still very complex [37]. Several researchers report that EGFR, Ras/MAPK, PI3K/Akt, Notch, Wnt/β-catenin, TGF-β, and Notch pathways all play important roles in the process of tumor drug resistance [38,39].

### 2.1. EGFR

EGFR is a tyrosine kinase encoded by the EGFR gene, which is widely expressed in normal tissues [40]. In recent years, epidermal growth factor receptor-tyrosine kinase inhibitors (EGFR-TKIs) have shifted from empirical cytotoxic chemotherapy to molecular-targeted therapy [41]. At present, three generations of EGFR-TKIs (gefitinib and erlotinib, dacomitinib and afatinib, and osimertinib) have been used in clinical practice and achieved positive results [42]. However, most patients develop drug resistance after 6–13 months of treatment with EGFR-TKIs [43]. It has been shown that molecular-targeted drugs such as EGFR-TKIs are in two different states: “on-target” and “off-target,” and the molecular mechanisms of tumor resistance to these states are different [44]. When the drug is in the “on-target” state, an EGFR mutation is the main cause of tumor resistance to the drug [45]. There are four members of the human EGFR receptor (HER) receptor tyrosine kinase family, EGFR (HER1 or ErbB1), HER2 (neu/ErbB2), HER3 (ErbB3), and HER4 (ErbB4) [46]. The EGFR mutations occur only on four exons, exons 18–21, with deletion, insertion, and missense point mutations. When exon 19 is deleted (Del19) or leucine (L) at position 858 of exon 21 is mutated to arginine (R) (L858R), impaired endocytosis of EGFR and increased tyrosine phosphorylation result [47]. The mutation of threonine (T) at position 790 in exon 20 to methionine (M) (T790M) is another important mutation commonly seen in clinical resistance to TKIs. Studies have shown that the T790M mutation causes acquired resistance to first- and second-generation TKIs in approximately 50–60% of patients [48]. Threonine 790 in exon 20 is the “gatekeeper” of the kinase structure, and when it is replaced by methionine, the EGFR kinase region undergoes conformational changes, rendering the TKI drug unable to access the active center of the tyrosine kinase and weakening reversible binding to the TKI drugs, which induces tumor resistance [49]. With advances in sequencing technology, scientists have also identified new mutation sites, such as L747S, D761Y, and T854A, which also reduce the binding interactions between the drug and EGFR-TKI to induce tumor resistance [50]. However, the mechanisms underlying drug resistance due to mutations remain unclear [51].

When the drug is in the “off-target” state, tumor resistance to the drug is primarily associated with signaling pathway interactions [52], such as between hepatocyte growth factor (HGF)/c-MET, vascular endothelial growth factor (VEGF), insulin-like growth factor 1 (IGF1), signal transducers and activators of transcription (STAT)3, and paracrine pathways [53]. c-MET is a receptor tyrosine kinase for a hepatocyte growth factor (HGF). It was found that 5–10% of lung cancer patients develop resistance to TKIs due to c-MET gene amplification [54]. This amplification, leading to ErbB3-dependent PI3K activation, contributes to tumor resistance to gefitinib [55]. HGF can induce resistance to gefitinib in lung adenocarcinoma patients with EGFR mutations by phosphorylating c-MET and activating the PI3K/Akt signaling pathway through an ErbB3-independent pathway [56]. HGF/c-MET also interacts with the Src/STAT3/FAK/ERK signaling pathway to induce resistance to platinum-based drugs in tumor cells [57]. Cancer-associated fibroblast-derived HGF can activate c-Met/PI3K/Akt and glucose-regulated protein 78 (GRP78) signaling pathways to promote tumor cell proliferation and induce drug resistance in tumor cells [58]. VEGF is an indispensable cytokine in angiogenesis, and VEGF signaling is mainly mediated by VEGF receptor 2 (VEGFR2). Studies have shown that in the microenvironment of interleukin (IL)-23-mediated tumor inflammation, the STAT3/VEGF pathway activates to induce resistance to doxorubicin in small-cell lung cancer (SCLC) cells [59]. IGF1 can promote tumor growth. The biological activity of IGF1 is primarily mediated by the IGF1 receptor (IGF1R). The IGF1R is a heterotetramer composed of tyrosine kinases located on the cell surface. The key factor in resistance to afatinib in NSCLC patients carrying the T790M mutation is the activation of the IGF1R signaling pathway [60]. Osimertinib-treated lung cancer patients were found to have increased IGF2 expression, and IGF2 autocrine-mediated IGF1R pathway activation is one of the causes of osimertinib resistance in lung cancer patients [61]. The STAT family is closely related to drug resistance production in NSCLC. The STAT3/zinc finger E-box-binding homeobox 1 (ZEB1) signaling axis was found to be a key factor in reversing resistance to gefitinib in NSCLC [62]. Long non-coding RNA MALAT1 positively regulates STAT3 and fructosyltransferase 4 (FUT4) activity and induces paclitaxel resistance in A549 lung cancer cells [63].

### 2.2. Ras/MAPK

Ras is a member of the small GTPase family and is the switch controlling GDP and GTP conversion. Functional mutations in Ras are one of the main causes of tumorigenesis [64]. The human Ras family includes four homologous Ras proteins: H-Ras, N-Ras, and two K-Ras splice variants (K-Ras4A and K-Ras4B), and are encoded by three RAS genes, HRAS, NRAS, and KRAS. Across all tumors caused by Ras mutations, K-Ras, N-Ras, and H-Ras mutations account for 85%, 12%, and 3%, respectively [64]. Studies have shown that oncogenic K-Ras mutations are closely associated with tumor resistance to platinum drugs [65]. In NSCLC, K-Ras mutations can induce nuclear factor-erythroid factor 2-related factor 2 (NRF2) gene transcription through TPE response elements, upregulating the NRF2 pathway, leading to excessive activation of an antioxidative stress pathway, reducing cisplatin-induced reactive oxygen species (ROS) generation in tumor cells, reducing the mortality of tumor cells, and therefore causing tumor cells to become resistant to cisplatin [66]. K-Ras mutations can also cause hyperactivation of the PI3K-AKT pathway, inhibit apoptosis, and increase cell proliferation [67].

Ras mutations are not only a cause of tumor resistance to platinum drugs, but also a common cause of tumor resistance to TKIs [68]. TKIs are widely used in the treatment of patients with clinical tumors, including renal cell cancer (RCC), colorectal cancer, acute myeloid leukemia (AML), and NSCLC [69]. The main mechanism of action of TKIs is to inhibit the phosphorylation sites within the protein, thus preventing them from exerting their kinase activity against downstream effectors [70]. Tumor resistance to TKIs occurs mainly by mutations in tyrosine kinases (RTKs) themselves or by mutations in downstream pathway proteins [71].

Tyrosine kinase 3 (FLT3) is very commonly mutated in AML, with FLT3 mutations present in approximately 40% of cytogenetically normal AML patients. Between 20 and 30% of patients with AML have FLT3-internal tandem duplication (ITD) mutations, and such patients have a high tumor recurrence rate and poor prognosis. Tumor cells can promote cell proliferation, inhibit apoptosis, and induce resistance to TKIs through the MAPK, STAT5, and PI3K pathways [72]. Therefore, scientists have developed many FLT3 inhibitors, such as gefitinib, clolani, and middolin, based on the active conformation of FLT3 [73,74]. Gefitinib and middoorin received FDA approval while Kolani is in a phase II trial [75,76]. Ras mutations can be detected in 30% of patients with resistance to gefitinib. Since Ras mutations can activate the MAPK and PI3K-AKT pathways, some tumor patients are also resistant to these novel FLT3 inhibitors [71].

### 2.3. PI3K/Akt

The PI3K/Akt/mTOR pathway is an important regulatory pathway for physiological activities such as cell proliferation, cell cycle, and apoptosis, and the hyperactivation of this signaling pathway plays an important role in cancer progression and cancer drug resistance [77]. Abnormal activation of the PI3K/Akt/mTOR pathway has been found to be one of the most important reasons for the development of tumor resistance to chemotherapy [78]. When Akt, the core protein of the PI3K/Akt/mTOR pathway, is activated by phosphorylation, activated Akt stimulates the serine/threonine-protein kinase PAK1, and PAK1 then upregulates the expression of the anti-apoptotic protein Bcl-2. PAK1 also promotes phosphorylation of Bad for release from the mitochondrial membrane to the cytoplasm and inhibits Bax transfer from the cytoplasm to mitochondria, attenuating the proapoptotic function of Bax and Bad, thus inhibiting tumor cell apoptosis and promoting resistance to chemotherapeutic drugs [79]. Tropicrine inhibited phosphorylation of Akt and resensitized multidrug-resistant breast cancer cells to chemotherapeutic drugs [80]. The activated PI3K/Akt pathway can then activate the NF-kB pathway, which results in increased intracellular protein synthesis, increased energy reserves, and rapid cell growth, favoring the formation of multidrug resistance in tumor cells [81]. Simultaneous activation of the PI3K/Akt/mTOR pathway and the MAPK signaling pathway can induce paclitaxel resistance in gastric cancer [82]. The mTOR mutation E2419K also contributes to tumor resistance to EGFR-TKI [83]. Studies have shown that the production of multidrug resistance in breast cancer cells is also closely related to Akt overexpression and phosphatase and tensin homolog (PTEN)/EMT/Akt activation [84,85]. Moreover, microRNA also modulates the PTEN/PI3K/Akt pathway to induce resistance of tumor cells to chemotherapeutic drugs [86]. For example, miR-132 and miR-212 regulate PTEN, leading to resistance to doxorubicin in breast cancer cells [87].

### 2.4. Wnt/β-Catenin

The Wnt signaling pathway is a very conserved pathway that is essential for cell fate and embryonic development in multicellular organisms. The Wnt genes are involved in cell proliferation, migration, apoptosis, and differentiation [88]. There are three main Wnt pathways: The β-catenin-dependent classical pathway, the planar cell polarity non-classical pathway, and the Wnt-Ca^2+^ non-classical pathway [89]. In the classical pathway, various factors leading to the massive accumulation of β-catenin in the cytoplasm and entry into the nucleus are key to activating Wnt/β-catenin signaling. Β-catenin promotes transcription of downstream target genes of the Wnt pathway [90]. The Wnt/β-catenin signaling pathway (classical Wnt pathway) is abnormally activated in tumor tissues and cells. The increased β-catenin activity was found to induce carboplatin resistance in A2780 cells [91]. However, the downregulation of β-catenin expression prevented β-catenin from entering the nucleus, which effectively increased the sensitivity of ovarian cancer cells to chemotherapeutic drugs and reversed resistance to platinum chemotherapeutic drugs [92]. Activation of the PI3K/Akt signaling pathway and an increase in nuclear β-catenin upregulated hypoxia-inducible factor 1 (HIF-1) expression and induced glucose metabolic reprogramming, conferring resistance to fluorouracil (5-FU) in colorectal cancer [93]. In colorectal cancer tissues, carnitine palmitoyltransferase 2 (CPT2) expression is downregulated, activating the ROS/Wnt/β-catenin pathway and inducing oxaliplatin resistance in colorectal cancer [94]. Xenograft tumor model studies from carboplatin-resistant breast cancer patients found that canonical Wnt/β-catenin signaling activation upregulated the expression of tumor stem cell markers and promoted tumor resistance to carboplatin. Inhibition of the Wnt/β-catenin signaling pathway restored the sensitivity of tumor tissue to carboplatin [95] and reversed the resistance to enzalutamide in prostate cancer [96]. Studies have shown that frameshift mutations in TCF7, a positive transcriptional regulator in the WNT/β-catenin signaling pathway, can induce abnormal WNT/β-catenin signaling, activate glycogen synthase kinase-3 (GSK3), and induce resistance to gedatolisib in colon cancer cells [97]. Moreover, small RNA can also regulate the Wnt/β-catenin signaling pathway to promote or inhibit the production of tumor resistance to chemotherapeutic drugs. For example, MiR-624-5p upregulates nod-like receptor family pyrin domain-containing protein 3 (NLRP3) expression through the EMT/IL-1/Wnt/β-catenin signaling pathway and promotes gemcitabine resistance in ovarian cancer [98]. MiR-331-3p activates Wnt/β-catenin through the suppressor of tumorigenicity 7 protein-like (ST7L) and promotes resistance to chemotherapeutic drugs in prostate cancer [99]. The targeting of miR-199b-3p to cysteine-rich transmembrane BMP regulator 1 (CRIM1) inhibits Wnt/β-catenin signaling and reverses resistance to cetuximab in colorectal cancer [100]. Both the ubiquitin-conjugating enzyme 2 proteins UBE2S and UBE2M can activate the Wnt/β-catenin signaling pathway, promoting resistance to olaparib in ovarian cancer and 5-FU resistance in colorectal cancer, respectively [101,102].

### 2.5. Notch

Notch signaling is a conserved ligand-receptor signaling pathway that plays a critical role in cell proliferation, survival, apoptosis, and differentiation, affecting the development and function of many organs [103]. Among the numerous targets of Notch are proteins that play a key role in tumor development and progression, such as the hairy and enhance of split (HES) family, hairy/enhancer of spit related with YRPW motif (HEY), nuclear factor-kappa B (NF-κB), VEGF, mTOR, cyclin D1, c-myc, p21, p27, and Akt [103].

Studies have shown that the Notch pathway regulates tumor stem cell formation, promotes the epithelial–mesenchymal transition (EMT), and participates in the formation of tumor resistance to chemotherapeutic drugs [104]. EMT plays an important role in the paclitaxel resistance process in cervical cancer. Notch1 expression was elevated in paclitaxel-resistant cervical cancer cells compared to non-resistant cells, and silencing the NOTCH1 gene reversed EMT, indicating that Notch1 is involved in the paclitaxel resistance process in cervical cancer [105]. Notch signaling is activated and is a key factor in developing resistance to endocrine therapy in estrogen receptor (ER)-positive (ER+) breast cancer. Endocrine therapy is the primary treatment modality in patients with ER+ breast cancer, and the main drugs are tamoxifen, aromatase inhibitors, and fulvestrant [106]. Tamoxifen and fulvestrant, competitive inhibitors of 17-estradiol, can directly bind to the ligand-binding domain of estradiol and inhibit 17-estradiol-mediated ER signaling in cancer cells. Aromatase inhibitors can reduce the aromatase-mediated local androgen synthesis of 17-estradiol, indirectly targeting the ER signaling pathway [107]. It was found that 17-estradiol-mediated estrogen synthesis maintains Notch activity at low levels, while antagonistic estrogen activates Notch signaling. Mutations in the ER hormone-binding domain, such as Y537S, can cause resistance to endocrine therapy in ER+ breast cancer [107]. Knockdown of Notch1 decreased cell proliferation and increased the sensitivity of prostate cells to benzalotamide, indicating that Notch1 signaling plays an important role in the production of enzalutamide resistance in prostate cancer, and inhibition of Notch1 signaling restored the sensitivity of resistant cells to enzalutamide [108].

### 2.6. TGF-β

Dysregulation of the TGF-β-signaling pathway leads to many diseases, including cancer. Although the activation of TGF-β signaling in healthy non-cancer and early cancer cells induced effective cell cycle arrest, elevated TGF-β expression and TGF-β receptor activation of intracellular signaling are observed in many cancers [109]. Cohort studies of tumor patients suggest that patients whose TGF-β pathway is activated in vivo have poor outcomes [110]. Activation of this pathway in tumor cells induces EMT [111]. In addition to its importance in migration, EMT has a role in resistance to chemotherapy [112,113]. TGF-β is mainly produced by tumor cells and stromal cells where it promotes tumor angiogenesis and therefore tumorigenesis. This suggests that TGF-β signaling changes during tumor progression from tumor suppression to tumor promotion. TGF-β has been found to induce tumor resistance to chemotherapy through multiple pathways [114]. For example, the miRNA-mediated activation of the TGF-β signaling pathway can increase DNA repair capacity, leading to tumor drug resistance [115]. Tumor cells undergoing autophagy upregulate E3 ubiquitin ligase and promote the degradation of SMAD7, thus activating TGFβ/SMAD signaling, inducing EMT, and promoting tumor cell proliferation and migration, which produces resistance to chemotherapeutics [116,117]. TGF-β/SMAD signaling activation directly induces G1 cell cycle arrest in tumor cells, leading to tumor proliferating cancer cells (TPCs) entering a quiescent state, protecting cancer cells from cellular DNA damage caused by 5-FU, and therefore inducing drug resistance [118]. In docetaxel-resistant and paclitaxel-resistant triple-negative breast cancer cells, aurora kinase A (AURKA) is highly expressed in the cells, where it mediates TGF-β-induced EMT and produces resistance to these drugs [119].

The immune system can accurately identify and remove malignant tumor cells. However, tumor cells alter or reduce the expression of tumor-specific antigens, upregulate immune checkpoint proteins, and alter the expression of cytokines to promote immune evasion [120]. TGF-β can inhibit the cytotoxicity and activation of immune cells, including macrophages, neutrophils, bone marrow-derived suppressor cells (MDSC), natural killer (NK) cells, and dendritic cells (DCs) and T cells, and inhibit immune cell function [121]. Tumor cells can secrete TGF-β, which directly induces the transformation of NK cells into innate lymphoid cell type 1 (ILC1) cells lacking cytotoxic function, inhibits the cytotoxicity mediated by the NK cell receptor NKG2D, promotes immune escape of tumor cells, and induces immune tolerance of tumor cells [121]. In the immunosuppressed microenvironment, glycoprotein A repetitions predominant (GARP) is highly expressed, which can induce the binding of dormant TGF-β to integrin αvβ8 on the cell membrane; the activated TGF-β is released, causing the immune escape of tumor cells [122]. Specific inhibition of TGF-β1 in Treg cells highly expressing GARP overcame drug resistance to programmed cell death protein 1 (PD1)/programmed cell death ligand 1 (PD-L1) blockers in tumor patients [123]. TGF-β also inhibits Th2 cells, which mediate tumor immunity. By blocking TGF-β signaling in CD4^+^ T cells, Th2 cells secrete IL-4, which reshapes the tumor immune microenvironment and suppresses tumor growth [124,125]. TGF-β1 can induce high expression of PD1 and PD-L1 in T cells and tumor cells, respectively, thus impairing the antitumor activity of T cells and promoting tumor immune evasion [126]. Although the major mechanisms of resistance to cancer immunotherapy have not been fully established, the inhibition of TGF-β signaling and modifying the tumor microenvironment may overcome tumor resistance to PD1\PD-L1 blockers [127,128].

## 3. Natural Products in Preventing Tumor Drug Resistance

### 3.1. Alkaloids

#### 3.1.1. Matrine

Matrine is a class of tetracyclic quinoline alkaloids extracted from plants in the genus *Sophora* with the formula C_15_H_24_N_2_O, and a molecular weight of 248.36 g/mol. The antitumor activity of matrine is mainly manifested by inhibiting cancer cell proliferation, blocking the cell cycle, inducing apoptosis, and inhibiting cancer cell metastasis. At the same time, matrine can reverse the resistance and reduce the toxicity of anticancer drugs [129]. Matrine and its compounds were reported to inhibit the growth and proliferation of various cancer cells through the induction of apoptosis and cell cycle arrest [130,131]. It can also inhibit tumor cell migration, invasion, and adhesion by downregulating the expression of certain active oncogenes [132,133]. Furthermore, it was found that the resistance reversal action of low doses of matrine can be improved by structural modification, such as the introduction of a thiophene structure in matrine [134]. Studies have found that matrine can reverse lung cancer [135], mammary cancer [80], and bladder cancer [136] resistance. Matrine can induce apoptosis in lung cancer cells by regulating the PI3K/AKT/mTOR signaling pathway, and simultaneously downregulating the expression of apoptosis inhibitory protein [135]. Matrine can also inhibit the expression of VEGF and the proliferation of breast cancer cells by regulating the Wnt/β-catenin signaling pathway [137]. Some matrine derivatives can also inhibit the proliferation of hepatocellular carcinoma (HCC) cells through the PI3K/AKT/mTOR and AKT/GSK3/β-catenin signaling pathway [138].

It was found that matrine exerts antitumor effects in drug-resistant ovarian cancer cells in vitro and in vivo by downregulating MAPK/ERK, PI3K/Akt, and Akt/mTOR signaling [139]. It shows a dose- and time-dependent growth inhibition by inducing increased G0/G1 and decreased S and G2/M phases in A2780 and SKOV3 cells, with a molecular mechanism associated with the upregulation of p21 and downregulation of cyclin D1 and CDK4 [140]. When studying the therapeutic effect of matrine on colorectal cancer (CRC), it was found that matrine could upregulate p21, p27, and pGSK-3 in a dose-dependent manner and downregulate CDK6, cyclinD1, and cyclin E, also in a dose-dependent manner [141]. Moreover, it has been found that nontoxic concentrations of matrine sensitized multidrug-resistant K562 cells in two ways, namely, the reactivation of apoptosis and inhibition of drug efflux [142]. Matrine can downregulate the phosphorylation of NF-κB, restore pro-apoptotic factors, and inhibit anti-apoptotic factors, thus promoting endogenous apoptosis [143]. Matrine can also induce mitochondrial damage by promoting the proapoptotic genes Bax, Bid, Bad, and Bim, and downregulating the apoptosis-inhibiting genes Bcl-2, Mcl-1, and Bcl-XL to promote endogenous Apoptosis. Additionally, matrine can downregulate ABCB1 expression, resulting in reduced drug efflux, which may also be related to the inhibition of NF-κB and facilitate the intrinsic apoptotic pathway by downregulating a downstream antiapoptotic factor Bcl-2 [80,142]. Finally, matrine significantly reversed the resistance of oxaliplatin-resistant HT-29/OXA cells with a dose-dependent increase in the sensitivity of HT-29/OXA cells to oxaliplatin [144]. Furthermore, it was shown that matrine combined with cisplatin inhibited urothelial bladder carcinoma (UBC) cells through the downregulation of the VEGF/PI3K/Akt signaling pathway. In the combination treatment, matrine improved the sensitivity of UBC cells to cisplatin and reduced the dose of cisplatin, thus potentially attenuating its side effects, suggesting that matrine may be a new option for UBC combination therapy [136].

#### 3.1.2. Tetrandrine

Tetrandrine (TET) is a class of dibenzyl isoquinoline alkaloids extracted from *Stephania tetrandra* with the molecular formula C_38_H_42_N_2_O_6_ and molecular weight of 622.76 g/mol. It mainly acts by regulating molecular signaling pathways, inducing apoptosis in cancer cells, promoting cell cycle arrest, and increasing cell autophagy. It has been reported that TET induced apoptosis at high concentrations and autophagy at low concentrations [145]. Studies have shown that it contributes to many types of cancer treatment, such as lung, breast, colon, and cervical cancer [146,147,148,149]. Although it exhibits mild side effects such as diarrhea, myelosuppression, and mucositis, it has been shown to be nontoxic to normal cells under in vivo experimental conditions. Otherwise, it did not change the pharmacokinetic parameters in in vivo assays, an advantage of its use over other compounds [145]. It was found that TET may block gefitinib-induced autophagic flow by inhibiting lysosomes and therefore enhance the sensitivity of PC14 cells to gefitinib. Because autophagy provides a large source of energy and material for cancer cell growth, the inhibition of autophagy may lead to cancer cell growth arrest [150]. Furthermore, TET can counter drug resistance and multidrug resistance in MDR1 gene-transfected cancer cells by downregulating ABCB1 transporter expression and consequently increasing the intracellular concentration of chemotherapeutic drugs [27].

TET was found to enhance SKOV3/paclitaxel cell sensitivity to paclitaxel by inducing apoptosis and cell cycle arrest in ovarian cancer therapy. Furthermore, TET could enhance the antitumor effects of paclitaxel in SKOV3/paclitaxel cells by inhibiting the β-catenin/c-myc/cyclin D1 signaling pathway [151]. In the treatment of pancreatic cancer, the powder promoted apoptosis and autophagy, thus restoring the chemosensitivity of gemcitabine-resistant human pancreatic cancer cells to gemcitabine. The proapoptotic effects of TET are associated with the reduction of survivin expression and downregulation of PI3K/Akt/mTOR signaling pathway activity [152]. In the treatment of gastric cancer, the alkali upregulated the caspase cascade proteins (cleaving PARP, caspase-3, and caspase-9) and inhibited the phosphorylation of Akt/mTOR, resulting in significant apoptosis of human gastric cancer cells [153]. In leukemia treatment, PPA induced apoptosis through caspase cascade regulation, cell cycle arrest, MAPK activation, and PI3K/Akt/mTOR signaling modification, showing cytotoxic effects on glucocorticoid-resistant human leukemia Jurkat T cells [154]. In bladder cancer treatment, TET induced autophagy in human bladder cancer cells by regulating AMPK/mTOR signaling, which favors apoptosis induction, suggesting that it may be a potential anticancer candidate for the treatment of bladder cancer [155].

#### 3.1.3. Ligustrazine

Ligustrazine (TMP) is an alkaloid monomer isolated from *Ligusticum xiong*. The formula is C_8_H_12_N2, with a molecular weight of 136.19 g/mol. However, ligustrazine is chemically unstable with a half-life of approximately 1.5 h, which partly limits its potential as a cancer therapeutic agent and, therefore, drug delivery systems compatible with it are being developed [156]. Previous studies have found that ligustrazine reduced the risk of multidrug resistance in chemotherapy and inhibited the proliferation and metastasis of various types of cancer cells, such as ovarian and liver cancer [157]. Studies have shown that ligustrazine can promote the antitumor effects of paclitaxel. Ligustrazine combined with paclitaxel inhibited angiogenesis by inhibiting the ERK1/2 and Akt pathways and promoted tumor cell apoptosis. Ligustrazine can decrease MRP1, GST, and BCL-2 to reverse multidrug resistance in human bladder cancer [158].

Furthermore, ligustrazine enhanced the antitumor effects of paclitaxel in vivo and somewhat attenuated the toxicity of paclitaxel [159]. It was found that K562 sensitivity and subsequent cytotoxicity of A02 cells (doxorubicin-resistant) in the presence of ligustrazine derivatives were enhanced due to doxorubicin accumulation in cells [160]. In addition, after ligustrazine and Danshensu (DSS) combine to form the conjugate DT-010, the complex reversed doxorubicin multidrug resistance in MCF-7/doxorubicin-resistant human breast cancer cells at non-cytotoxic concentrations. This effect may be related to P-gp inhibition [161]. Finally, it has been shown that ligustrazine suppresses retinoblastoma growth by regulating CXCR4 expression, and it has been established that this effect is related to cell density. Therefore, ligustrazine is as a potential drug candidate for the treatment of retinoblastoma [162].

#### 3.1.4. Neferine

Lotus heart base is a dibenzylisoquinoline alkaloid isolated from the green seed embryo of the lotus flower. The formula is C_38_H_44_N_2_O_6_, and the molecular weight is 624.778 g/mol. A very important feature of neferine activity is that it is cytotoxic to hepatoma cells but does not damage normal human hepatocytes. Neferine can effectively inhibit the proliferation of multidrug-resistant cancer cells and induce autophagy [163]. Previous studies have shown that the lotus heart base has certain efficacy in many types of cancer, such as lung cancer [164], neuroblastoma [165], and ovarian cancer [166]. Pretreatment with neferine can activate the MAPK/mTOR pathway to combat cisplatin-induced apoptosis and regulate autophagy [167]. Some studies have reported that neferine activates the Ryanodine receptor through the AMPK/mTOR-dependent pathway, thus inducing autophagy and releasing calcium ions [168]. It has been demonstrated that neferine can inhibit angiogenesis in ovarian cancer by inhibiting the mTOR/p70S6K signaling pathway while inducing autophagy and inhibiting M2 macrophage polarization [166].

In addition, it was found that the natural alkaloid neferine can promote apoptosis and induce the cell cycle by inducing JNK and p38 MAPK phosphorylation and downregulating PI3K and NF-κB signaling, enabling CD44^+^, and therefore inhibiting the proliferation and migration of CSCs. Neferine treatment not only inhibited CD44^+^ CSC activity, but it also inhibited the survival of androgen-insensitive PC3 and androgen-sensitive LNCaP cells. This suggests that neferine may be eradicating prostate cancer (PCa) cells and CD44^+^ CSC and may inhibit metabolism. By acting on CSC, it can effectively combat the development of drug resistance and disease recurrence [169]. Mitomycin C is a known antitumor drug that, upon binding to neferine, continuously activates the p38 MAPK pathway in a ROS-dependent manner, thereby inducing apoptosis in cervical cancer cells [170]. It also induced G1 phase growth arrest in osteosarcoma cells, Hep3B cells, and adenocarcinoma cells, and induced autophagy in PC-12 cells, human ovarian cancer cells, and Hep3B cells. The mechanisms inducing growth arrest are increased stability of p21 and phosphorylation at Ser130, whereas autophagy induction involves the inhibition of PI3K/Akt/mTOR signaling in A549 cells, and the activation of p38 MAPK/JNK in human ovarian cancer cells. Studies have shown that neferine cooperates with anticancer compounds (such as doxorubicin, paclitaxel, cisplatin, and vincristine) in many cancer cells, thus improving efficacy and combating chemotherapy resistance. Neferine combined with isoliensinine could downregulate the cell survival protein expression (PI3K/pAkt/mTOR) and activate mitochondria-mediated apoptosis by upregulating Bax, cytochrome c, caspase-3, and PARP cleavage expression while downregulating BCl-2 expression in cisplatin-resistant colon stem cells (CSCs) [171]. In addition, neferine can enhance the sensitivity of some types of cancer cells to chemotherapeutic drugs and reduce epithelial–mesenchymal transitions (EMT) due to chemoresistance against liver cancer cells. This fully illustrates the resistance reversal activity of neferine [172].

#### 3.1.5. Dauricine

Dauricine is a dibenzylisoquinoline alkaloid found in pueraria with the formula C_38_H_44_N_2_O_6_ and a molecular weight of 624.77 g/mol. It has potent antitumor activity, including the induction of apoptosis and overcoming drug resistance of tumor cells. Dauricine was found to reduce the survival of renal cell carcinoma (RCC) cells and induced cell cycle arrest in the G0/G1 phase and induced apoptosis through the intrinsic pathway [173]. Among these mechanisms, the former was associated with the downregulation of cyclin D1, CDK2, and CDK4 and the upregulation of p21. The latter was associated with the activation of caspase-9, caspase-3, and the downregulation of anti-apoptotic Bcl-2 protein expression. Moreover, dauricine also inhibited PI3K/Akt signaling activation and exerted antitumor effects [174]. In addition, it inhibited melanoma cell proliferation and strongly induced melanoma cell death by inhibiting the phosphorylation and activation of the Src protein and the activation of downstream signals (e.g., STAT3 protein) [175]. Dauricine downregulated the expression of hexokinase 2 (HK2) and pyruvate kinase M2 (PKM2). HK2 and PKM2 can be directly targeted by miR-199a. Dauricine dose-dependently increased miR-199a expression in HCC cells. This means dauricine suppressed glycolysis through miR-199aHK2/PKM2, increasing the sensitivity of hepatoma cells to chemotherapeutic drugs [176].

#### 3.1.6. Cepharanthine

Cepharanthine is an alkaloid extracted from *Stephania* with the formula C_37_H_38_N_5_O_6_, and a molecular weight of 606.71 g/mol. It exhibits multiple antitumor pharmacological effects, such as the induction of apoptosis, inhibition of angiogenesis, metastasis, and reversal of multidrug resistance [177,178,179]. Cepharanthine can induce autophagy by inhibiting Akt/mTOR signaling in human breast cancer MCF-7 and MDA-MB-231 cells, and stimulating AMPK-mTOR-dependent autophagy to induce apoptosis-tolerant cell death [180]. Furthermore, cepharanthine can induce apoptosis not only by upregulating the expression of initiator caspases such as caspase-8 and 9, but also by increasing the expression of effector caspases such as caspase-3 and 6 through caspase cascade regulation. Cepharanthine also activates MAPK and modifies PI3K/Akt/mTOR signaling pathway to show cytotoxic effects on glucocorticoid-resistant leukemic Jurkat T cells by increasing the phosphorylation of JNK and p38 [154]. It has been shown that cepharanthine hydrochloride can reverse P-gp-mediated multidrug resistance in A2780/paclitaxel cells. Furthermore, the mechanism by which galentin hydrochloride induced the reversal of multidrug resistance in human ovarian cancer may be related to the inhibition of the PI3K/Akt signaling pathway [181]. Mutant p53 is a major reason for the ineffectiveness of many anticancer drugs. It has been shown that cepharanthine alone in vitro and in vivo or in combination with 5-FU effectively controlled the growth of HT-29 human colorectal cancer cells carrying mutant p53. Cepharanthine with 5-FU also induced apoptosis and significantly upregulated BAK and cleaved PARP expression in tumor tissues. At the same time, cepharanthine prevented 5-FU-induced breast cancer drug-resistant protein and multidrug resistance-associated protein 1 (MRP1) expression [182].

#### 3.1.7. Solanine

Solanine is a type of toxic steroidal glycoside alkaloid. It is a toxic substance produced by potatoes after germination, and when they become green or ulcerated. It can exert anticancer effects by inhibiting cell proliferation, inducing apoptosis, blocking the cell cycle, inducing autophagy, enhancing chemoradiation, inhibiting epithelial–mesenchymal transformation, inhibiting tumor metastasis, and inhibiting angiogenesis, such as in liver cancer, breast cancer, liver cancer, pancreatic cancer, and colorectal cancer [183]. Studies have shown that serine inhibits the growth of transplanted hepatocellular carcinoma (HCC) in mice and reduces the CD4^+^CD25^+^Foxp3^+^ proportion of Treg and the expression levels of Foxp3 and TGFβ mRNA, and enhanced the body’s antitumor immune response by inhibiting the TGF/SMAD signaling pathway [184]. Furthermore, it reduced the expression and activity of Akt and ER, and this inhibitory effect may contribute to inactivated PI3K/Akt and ER signaling pathways in human endometrial cancer cells [185]. In addition, solanine enhanced the sensitivity of esophageal cancer cells to chemotherapy through the miR-138/survivin pathway [186].

### 3.2. Flavonoid

#### 3.2.1. Quercetin

Quercetin is a flavonoid, widely found in the flowers, leaves, and fruits of many plants, such as onions, apples, and hawthorn. The formula is C_15_H_10_O_7_ with a molecular weight of 302.24 g/mol. Quercetin can exert anticancer effects in various ways, such as through its antioxidant, antiproliferative, cell cycle arrest, apoptosis induction, and inhibition of migration effects [187]. It was found that quercetin was able to reverse drug resistance in paclitaxel-resistant prostate cancer cells in vitro by reversing the activation of the androgen receptor and PI3K/Akt signaling pathway in prostate cancer treatment [28]. Quercetin could significantly increase PI3K/AKT/mTOR axis and endurance gemcitabine-induced cytotoxicity in MIA Paca-2 and MIA Paca-2^GEMR^ cells [188]. In the treatment of colon cancer, when quercetin was combined with ionizing radiation (IR) therapy, colon cancer stem cells as target cells can have antitumor effects by inhibiting Notch-1 signaling [189]. Alternating consumption of quercetin and beta-glucan has been reported to reduce mortality in a colon cancer mouse model [190]. In addition, the combination of quercetin and heparin-bound cytokine (MK) can significantly promote apoptosis by downregulating the expression of PI3K/PTEN, MAPK, and NF-B signaling, leading to G1 cell cycle arrest and the inhibition of PC3 and CD44^+^/CD133^+^ cell migration, thus effectively eliminating cancer and tumor CSCs. Furthermore, the downregulation of MK expression with quercetin binding limits CD44^+^/CD133^+^ migration and the development progression of tumor cells as well as PCa cells [191]. It has been shown that quercetin can enhance the efficacy of the chemotherapeutic agents BEL/5-FU with ABCB1, ABCC1, and ABCC2 overexpression by blocking FZD7/β-catenin signaling. Thus, quercetin can act as an MDR reversal agent mediated by ABCB1 or ABCC1/2 [192].

#### 3.2.2. Curcumin

Curcumin is an Indian dietary polyphenol derived from turmeric roots, which includes three major bioactive components, namely curcumin, demethoxy curcumin (DMC), and didemethoxycurcumin at a ratio of 77:17:3. The molecular formula is C_21_H_20_O_6_ with a molecular weight of 368.39 g/mol. Curcumin is a highly potential alternative therapy for lung cancer with fewer side effects. It can exert its anticancer effects in lung cancer by modulating various molecular targets, signaling pathways, epigenetic alterations, and microRNA expression. However, the bioavailability of curcumin is relatively low, and its clinical application is limited to some extent [193]. In HCC treatment, curcumin enhanced the chemosensitivity of HCC cells to 5-FU in vitro, increased the apoptosis rate, arrested the cell cycle in the G2/M phase, and blocked PI3k/AKT/mTOR signaling by inhibiting the phosphorylation of PI3k and its downstream protein kinase. Curcumin also significantly sensitized H22 cells to 5-FU, enabling it to inhibit tumor growth in vivo [194]. In lung cancer treatment, when the PI3K-Akt pathway is inactivated, curcumin (DMC) enhanced the sensitivity of drug-resistant lung cancer cells to cisplatin by downregulating the expression of thymidine phosphorylase and RCC1 [195]. In addition, Didemethoxylated curcumin may increase the sensitivity of cisplatin-resistant lung cancer cells to chemotherapy by inhibiting CA916798 (an over-expressed MDR protein) and PI3K/AKT/mTOR signaling, thus exerting antitumor activity and reversing MDR [196]. Studies have shown that curcumin can induce apoptosis by reducing Akt and mTOR phosphorylation, thereby inhibiting the PI3K/Akt/mTOR pathway in A549 and H1299 NSCLC cells [197,198,199]. In colorectal cancer treatment, curcumin can reverse oxaliplatin resistance by inhibiting TGF-β/Smad2/3 signaling in vitro and in vivo [200]. Furthermore, HER2 is a transmembrane receptor with tyrosine kinase activity associated with cell proliferation, survival, metastasis, and drug resistance, and its overexpression is frequently seen in various types of human cancers, such as bladder cancer. DMC can induce apoptosis in bladder cancer cells with HER2 overexpression, via the degradation of HER2 and inhibition of the PI3K/Akt pathway [201]. In glioma therapy, the miR-145/SOX2-Wnt/β-catenin axis plays a key role in DMC-mediated glioma stem cell (GSC) inhibition, and upregulation of miR-145 can effectively enhance the effect of DMC against GSC; therefore, this may be a new therapeutic target for GSC resistance [202].

### 3.3. Terpene

#### 3.3.1. Ginsenoside

Ginsenoside is a triterpenoid isolated from ginseng that can exert antitumor effects through various mechanisms, including the induction of apoptosis, inhibition of proliferation, metastasis, angiogenesis, and activation of immunity [203]. The use of ginsenoside Rg3 may contribute to reducing toxicity and improving chemosensitivity in cancer combination therapy [204]. It was found that ginsenoside Rg3 reduced cisplatin resistance by upregulating miR-429 to inhibit the SOX2 and PI3K/Akt/mTOR signaling axes in gastric cancer treatment [30]. In pancreatic cancer treatment, ginsenoside Rg3 regulated the survival of pancreatic cancer cells via the PI3K/Akt/mTOR pathway [205]. In osteosarcoma treatment, ginsenoside Rg3 inhibited the proliferation and migration of osteosarcoma cells and induced apoptosis by reducing the protein expression of Bcl 2 and the PI3K/AKT pathway [206]. In glioblastoma treatment, ginsenoside Rg3 inhibited O6-methylguanine-DNA-methyltransferase (MGMT) by regulating the Wnt/β-catenin pathway and significantly enhanced the sensitivity of glioma to TMZ chemotherapy. Additionally, we found that 20(S)-ginsenoside-Rg3 significantly inhibited the epithelial–stromal transition process in glioma cells [207]. It was found that in taxol-resistant human nasopharyngeal carcinoma cells, ginsenosides Rg1 exerted antitumor activity by activating autophagic cell death, apoptosis, endogenous ROS production, S phase cell cycle arrest, and inhibition of the m-TOR/PI3K/AKT signaling pathway [208]. In addition, ginsenoside Rk1 reduced cell viability and colony formation, and triggered lactate dehydrogenase (LDH) leakage, G0/G1 phase arrest, and apoptosis. Furthermore, the ROS/PI3K/Akt signaling pathway was involved in Rk1-induced MDA-MB-231 cell death. These results suggest that ginsenoside Rk1 may be a potential antitumor agent for triple-negative breast cancer [209].

#### 3.3.2. Soil Bastard Saponin

Soil bastard saponin (TBM) is a terpenoid isolated from *Porcini*, whose main pharmacological active components are triterpenoid saponins, including TBM-I, II, and III. TBMs can inhibit cell growth and proliferation through various signaling pathways, such as ROS/cytochrome C/caspase-3, ROS/MAPK, MAPK-JNK, MAPK/p38, PI3K/Akt/mTOR, p53/MDM2, NF-κB, VEGF-A/VEGFR-2/ERK, MEK/ERK, Wnt/β-catenin, CXCL12-CXCR4, and Akt-mTOR-eEF-2K to induce cell differentiation, apoptosis, and autophagy, and inhibit inflammation, angiogenesis, invasion, and metastasis. Combination treatment with earth shellfish mother saponin has shown curative effects for drug-resistant colorectal cancer cells (CRC) [210,211].

## 4. Summary

Most of the research on cancer drug-resistance mechanisms is displayed in experimental animals. However, drug resistance at the cell level has not been confirmed in the clinic. Tumor multi-drug resistance pathogenesis is complex, so the actual clinical significance of many mechanisms is unclear. The best means to study tumor drug resistance is to obtain human tumor tissue for research. In recent years, high-throughput technology in China has made great progress in the field of biomedicine, using high-precision technology, combined with modern pharmacology, pharmacodynamics, molecular biology, and other new technology, from the single-cell level of tumor molecular mechanisms.

However, natural products show some problems, such as poor solubility, poor permeability, low bioavailability, instability in biological milieu, and extensive first-pass metabolism in drug delivery systems. Some delivery strategies, exosomes or nanotechnology, have attempted to overcome these limitations, rediscovering new benefits associated with these natural products. Exosomes or nanotechnology can certainly enhance the pharmacokinetics and therapeutic index of natural active natural products and improve their performance in therapy.

**Figure 1:**
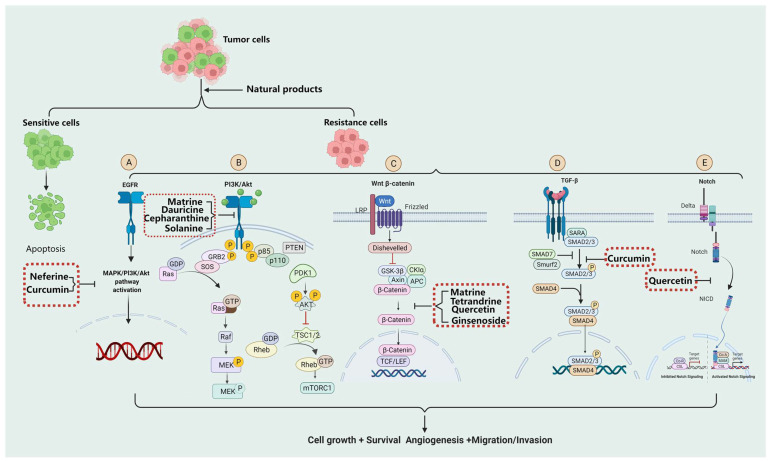
Molecular mechanisms of tumor drug resistance induced by EGFR, PI3K/Akt, Wnt/β-catenin, TGF-β, and combinations of these signaling pathway inhibitors’ derivatives from natural products to overcome tumor resistance. (**A**) EGFR signaling pathway and its inhibitors’ derivatives from natural products. (**B**) PI3K/Akt signaling pathway and its inhibitors derivatives from natural products. (**C**) Wnt/β-catenin signaling pathway and its inhibitors’ derivatives from natural products. (**D**)TGF-β signaling pathway and its inhibitors’ derivatives from natural products. (**E**) Notch signaling pathway and its inhibitors’ derivatives from natural products.

Chinese medicine application has a long history in China, with rich resources and pharmacological effects. We reviewed the signaling pathways involved in the development of tumor drug resistance, and their specific signaling pathway inhibitors derived from natural products (Figure 1). This provides a theoretical basis for drug resistance and expanding the range of candidate compounds to all natural products, including traditional Chinese medicine (TCM) extracts, TCM compound preparations, and derivatives, in order to find new and efficient tumor multidrug resistance treatments using a new direction, bringing a new dawn.

## Figures and Tables

**Table 1:** Natural products in preventing tumor drug resistance.

Natural Products	Molecular Structure	Cancer	Signaling Pathway
1. Alkaloids			
Matrine	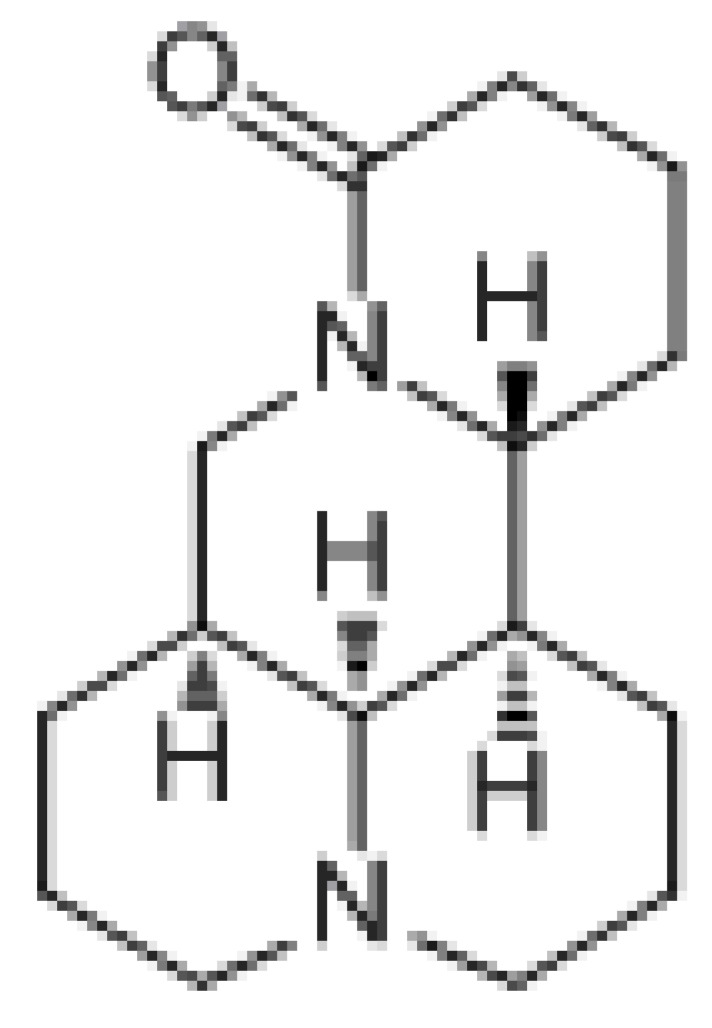	Lung cancer	PI3K/AKT/mTOR
		Breast cancer	Wnt/β-catenin
		Hepatocellular carcinoma	PI3K/AKT/mTOR, AKT/GSK3/β-catenin
		Ovarian cancer	MAPK/ERK PI3K/AKT/mTOR
		Urothelial bladder carcinoma	VEGF/PI3K/AKT
Tetrandrine	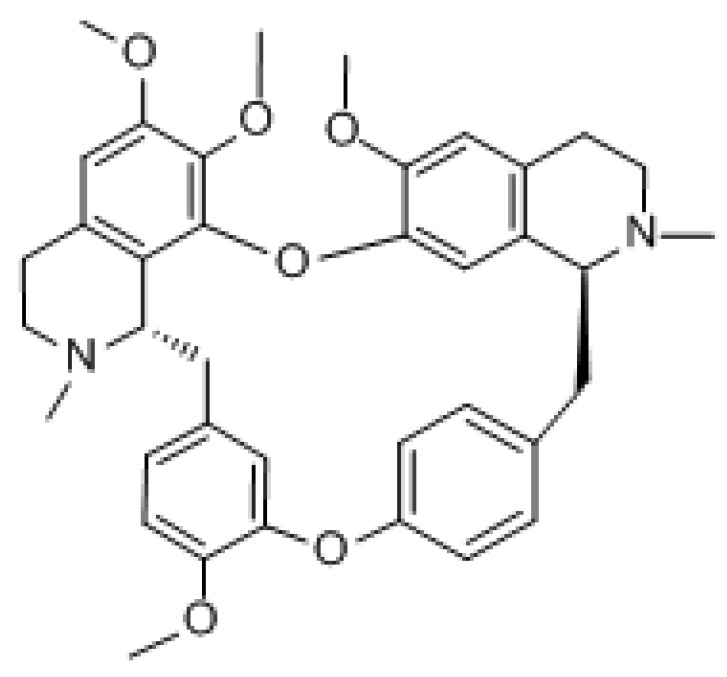	Ovarian cancer	β-catenin/c-myc/cyclin D1
		Pancreatic cancer	PI3K/AKT/mTOR
		Gastric cancer	AKT/mTOR
		Bladder cancer	AMPK/mTOR
Neferine	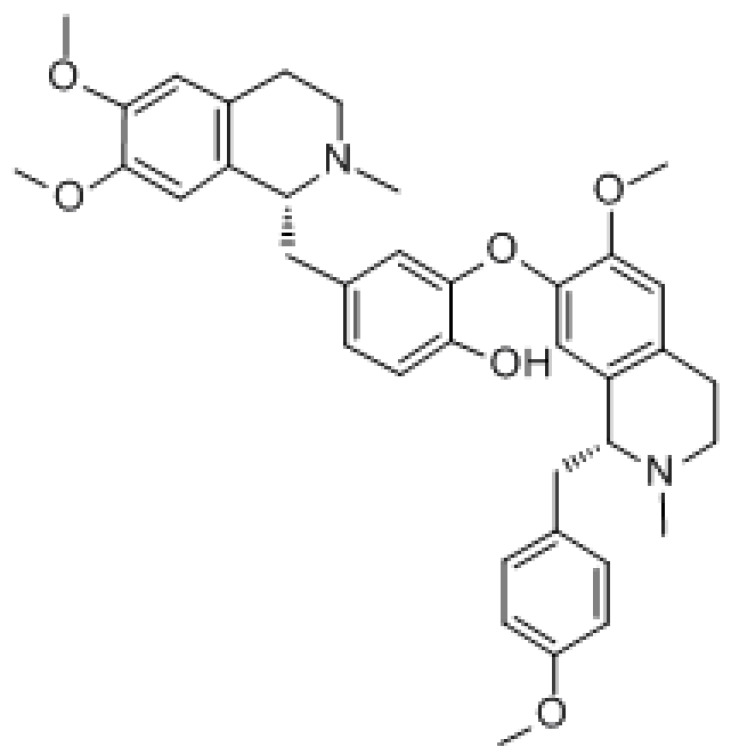	Ovarian cancer	mTOR/p70S6K, MAPK/JNK
		Lung cancer	PI3K/AKT/mTOR
Dauricine	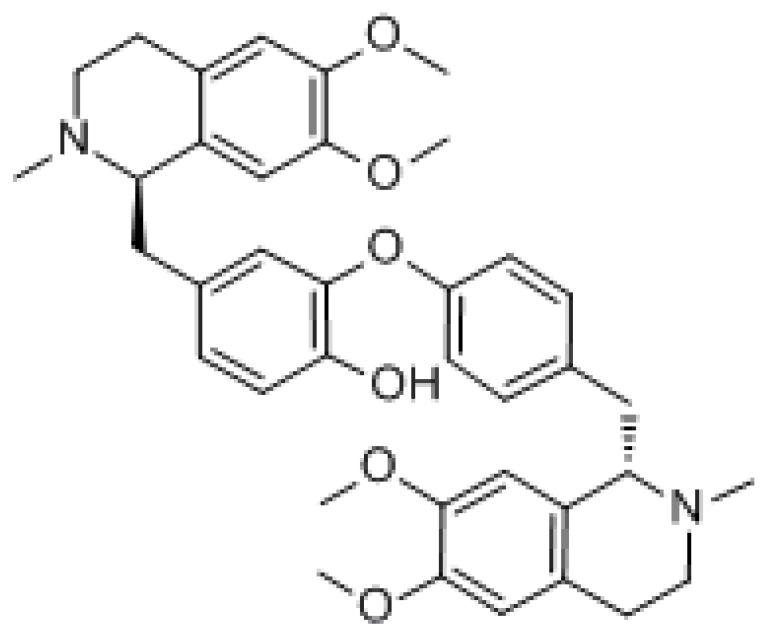	Renal cell carcinoma	Cell cycle
		Melanoma	Src/STAT3
		Hepatic cell carcinoma	miR-199aHK2/PKM2
Cepharanthine	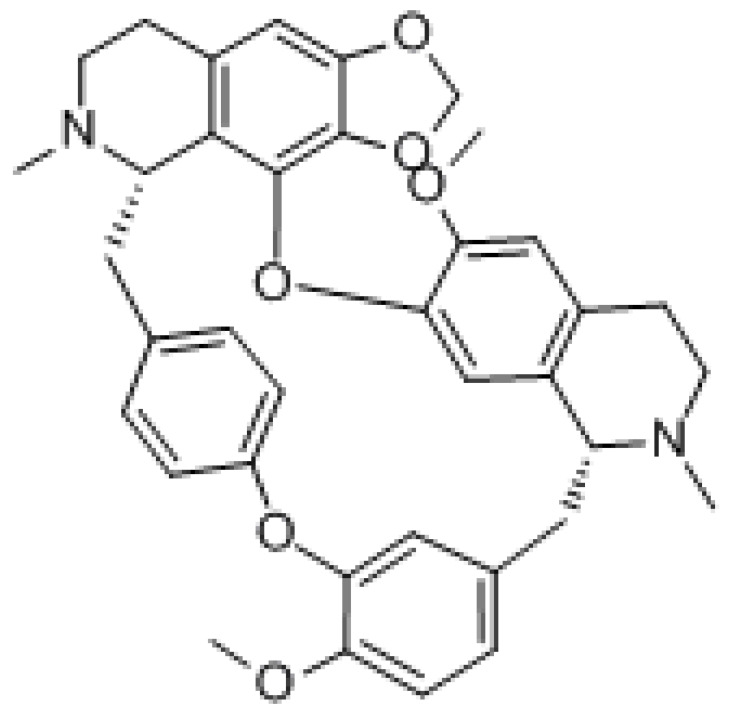	Breast cancer	Akt/mTOR
		Ovarian cancer	PI3K/AKT
Solanine	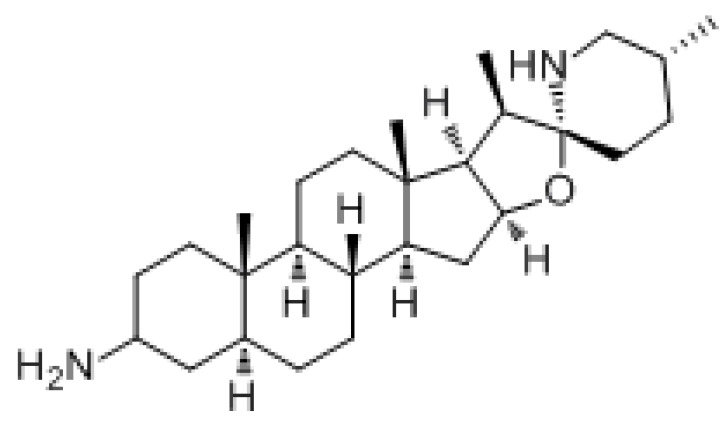	Endometrial cancer	PI3K/AKT
2. Flavonoid			
Meletin	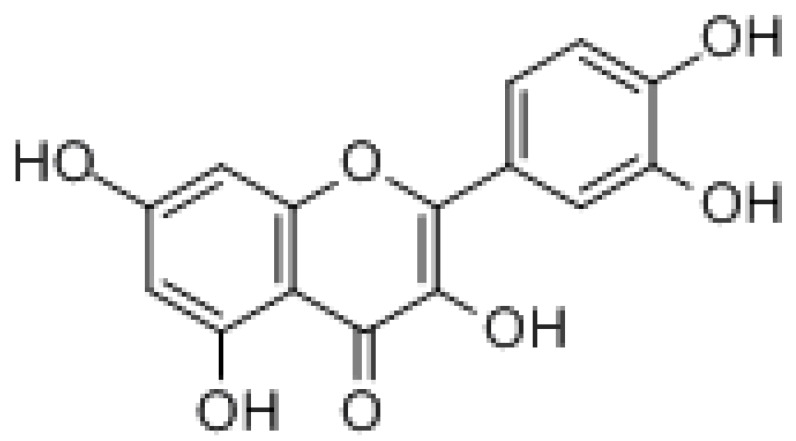	Prostate cancer	PI3K/AKT
		Colon cancer	Notch-1
Curcumin	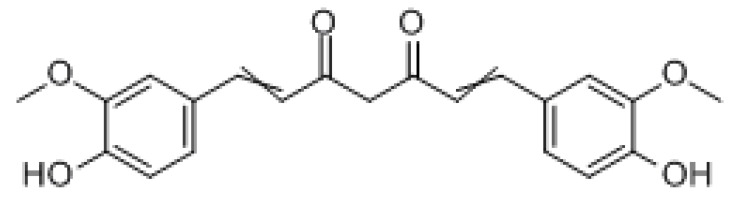	Hepatocellular carcinoma	PI3K/AKT/mTOR
		Lung cancer	PI3K/AKT
		Colorectal cancer	TGF-β/Smad2/3
3.Terpene			
Ginsenoside	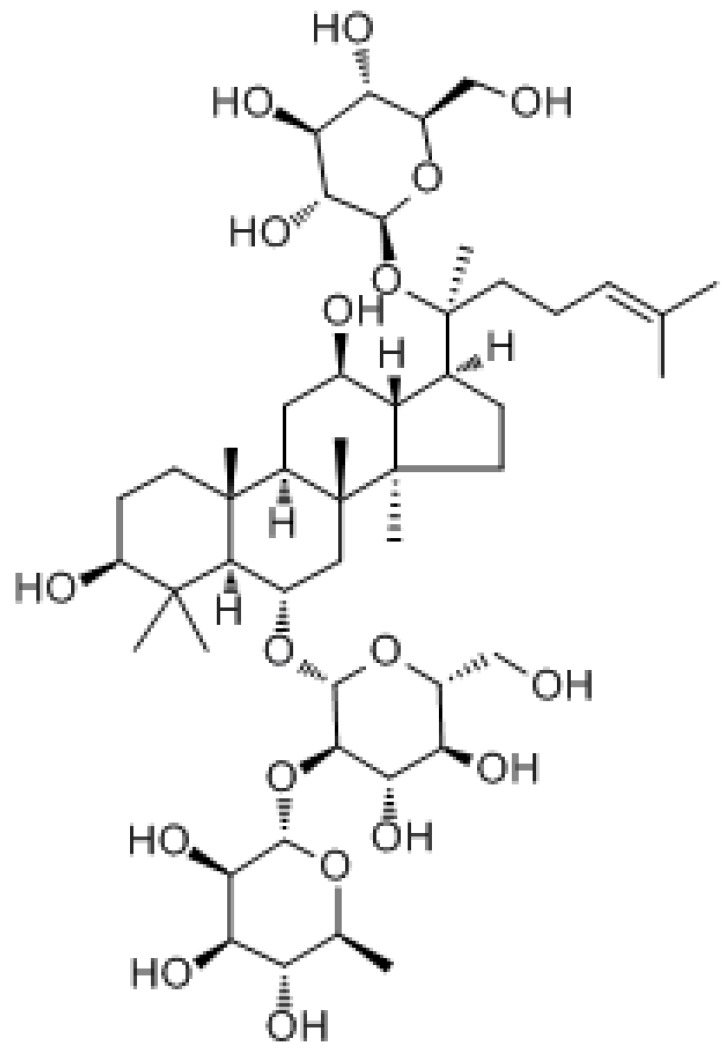	Gastric cancer	PI3K/AKT/mTOR
		Glioblastoma	Wnt/β-catenin
Soil bastard saponin	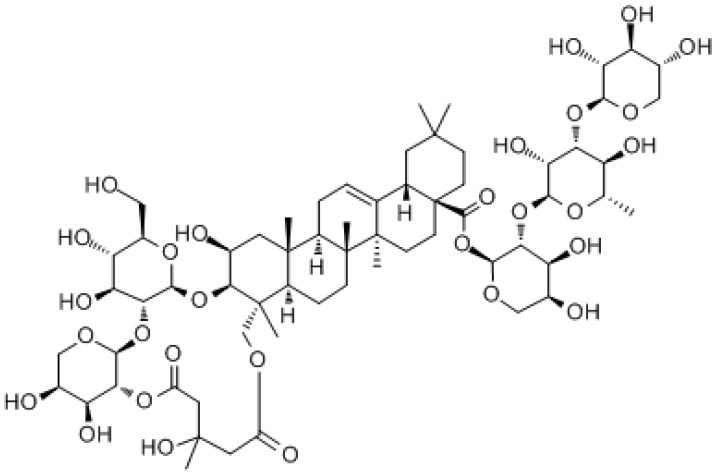	Colorectal cancer	Wnt/β-catenin signaling pathway

## Sample Availability

Samples of the compounds curcumin are available from the authors.
